# Fabrication of Algal Polysaccharides-Based Nanoparticles and Evaluation of Their Antioxidant and Anti-Inflammatory Potential

**DOI:** 10.1049/nbt2/8887357

**Published:** 2025-11-20

**Authors:** Cheng-Yuan Chen, Wei-Hao Huang, Pin-Yu Tsai, Chung-Hsiung Huang

**Affiliations:** ^1^Department of Food Science, National Taiwan Ocean University, Keelung, Taiwan; ^2^Center for Marine Bioscience and Biotechnology, National Taiwan Ocean University, Keelung, Taiwan

**Keywords:** algal polysaccharides, anti-inflammation, antioxidation, dry-heating, nanoparticles

## Abstract

This study investigates the development of nanoparticles derived from algal polysaccharides and evaluates their physicochemical properties, antioxidant capacity, and anti-inflammatory activity in comparison to their native counterparts. Polysaccharides extracted from *Sargassum* (SP), *Ulva* (UP), and *Porphyra* (PP) were subjected to dry-heating at various temperatures to form nanoparticles. The prepared polysaccharides and nanoparticles were characterized by molecular weight distribution, monosaccharide composition, yield, morphology, particle size, sulfate content, and functional group profiles, respectively. The nanoparticles were spherical in form, with diameter less than 500 nm. Furthermore, their polydispersity index (PDI) was observed to be lower than 0.4, and their zeta potentials ranged from −5 to −30 mV. Dry-heating above 210°C induced notable alterations in functional groups, while temperatures above 150°C significantly enhanced DPPH radical scavenging and Fe^2+^ chelation activities. The nanoparticles showcased enhanced antioxidant and anti-inflammatory capabilities when juxtaposed with crude polysaccharides. Specifically, they led to a significant suppression of lipopolysaccharide (LPS)-induced generation of key pro-inflammatory molecules in macrophages. Importantly, the nanoparticles exhibited no cytotoxicity at concentrations below 1000 *μ*g/mL. These findings suggest that algal polysaccharide-based nanoparticles, particularly those formed at higher temperatures, hold considerable potential as bioactive agents in therapeutic applications.

## 1. Introduction

The inflammatory response is a protective mechanism triggered by external stimuli or tissue damage, leading to the activation of macrophages at the affected site. These macrophages engage in phagocytosis and release key mediators, which contribute to the inflammatory process [1]. Oxidative stress happens when the amount of free radicals produced overwhelms the antioxidant defense system, commonly occurring alongside inflammation. This imbalance can aggravate tissue damage and is a key contributor to various chronic diseases [2]. RAW 264.7 cells serve as a widely utilized macrophage model in studies exploring the mechanisms and efficacy of anti-inflammatory agents, as they respond robustly to lipopolysaccharides (LPSs), a potent inducer of inflammation [3]. LPS stimulation triggers pathogen-recognition receptors, which in turn initiate signaling cascades that upregulate the expression of pro-inflammatory mediators, exacerbating the inflammatory response [4]. TNF-*α*, IL-1*β*, and IL-6 are major pro-inflammatory cytokines that play essential roles in immune responses. However, their sustained overproduction is linked to the development and persistence of chronic inflammation [5–7]. Nitric oxide, produced by macrophages in response to cytokines, further exacerbates inflammation when present in excess [8]. Additionally, oxidative stress can drive lipid peroxidation, generating free radicals that worsen inflammation, whereas antioxidants help mitigate this damage by neutralizing these reactive species [9]. Antioxidant activity is often measured through the DPPH assay, which evaluates the ability of compounds to inhibit oxidation [10]. The ability to chelate metal ions, particularly Fe^2+^, is another important antioxidant mechanism [11].

Algae have long been used as food sources in various Asian cultures, with over 200 edible species documented. Among these, brown, red, and green algae are particularly prominent [12]. Algae are rich in carbohydrates, with total carbohydrate content ranging from 5% to 75%, depending on species, growth conditions, and harvesting time. These carbohydrates are predominantly polysaccharides, recognized for their demonstrated antioxidant and anti-inflammatory attributes [13]. For example, polysaccharides from *Sargassum* (SP), a type of brown algae, have demonstrated anti-inflammatory effects by reducing cytokine levels and nitric oxide production in LPS-induced inflammation models [14]. Similarly, polysaccharides from *Ulva* (UP), a type of green algae, have exhibited antiviral, antioxidant, and immunomodulatory activities and can alleviate allergic inflammation [15, 16]. Polysaccharides from *Porphyra* (PP), a type of red algae, possess antioxidant property and can modulate immune responses to reduce intestinal inflammation [17, 18]. These studies underscore the therapeutic potential of algal polysaccharides in managing inflammation. The bioactivity of algal polysaccharides is largely determined by their structural characteristics, such as molecular weight and specific functional groups; however, their limited oral bioavailability poses a significant challenge to practical application [19].

Nanomaterials, particularly those at the nanoscale, possess unique biological activities due to their large surface area and structural precision, which significantly influence their interactions with biological systems [20]. Among them, carbon-based nanomaterials have gained considerable attention for their excellent biocompatibility and promising biomedical applications [21]. Notably, nanotechnology offers a powerful strategy to overcome the limitations of algal polysaccharides, such as poor oral bioavailability and instability during gastrointestinal transit. By converting these polysaccharides into nanoparticles, it is possible to enhance their solubility, stability, targeted delivery, and overall bioavailability [21]. The dry-heating method is a simple, solvent-free, and cost-effective approach for preparing such nanomaterials. By adjusting heating temperature and duration, this method can modify surface functional groups and physicochemical properties, resulting in nanoparticles with improved water solubility, reduced toxicity, and enhanced biocompatibility [22]. These advantages make the dry-heating technique a promising strategy for developing bioactive nanoparticle formulations from algal polysaccharides.

This study presents the first investigation into the preparation of nanoparticles solely from SP, UP, and PP polysaccharides using a dry-heating method. While algal polysaccharides are known for their antioxidant and anti-inflammatory properties, no prior research has explored their transformation into nanoparticles via this simple approach. Here, we evaluate whether dry-heating these polysaccharides at various temperatures enhances their bioactivity, aiming to determine if the nanoparticles exhibit stronger antioxidant and anti-inflammatory effects than the unprocessed algal polysaccharides.

## 2. Materials and Methods

### 2.1. Algal Sources, Chemicals, and Assay Kits

SP and PP were obtained from Professor Jui-Sheng Chang (National Taiwan Ocean University, Keelung, Taiwan) and Pin Jia Biotechnology, Ltd. (Pingtung, Taiwan), respectively. UP was purchased from Sweet Town Enterprise Corp. (Hualien, Taiwan). All chemicals, reagents for cell culture, and kits were obtained from Sigma–Aldrich (St. Louis, MO, USA), Panreac (Barcelona, Spain), Corning Inc. (New York, NY, USA), and Invitrogen (Waltham, MA, USA).

### 2.2. Preparation and Characterization of Algal Polysaccharides and Algal Polysaccharides-Based Nanoparticles

Polysaccharides were extracted from SP and PP following previously reported methods [18, 23]. In brief, 25 g of algal powder were combined with 1 L of deionized water. Afterwards, the mixture underwent heating at 121°C for 15 min. After cooling, the solution underwent centrifugation at 4°C and 12,000 × *g* for a period of 20 min. The resulting supernatant was then precipitated using 95% ethanol in a 1:6 volume ratio, and the mixture was incubated for 24 h before a final centrifugation step. The pellet was vacuum lyophilized to obtain crude polysaccharides. The extract was dissolved in 1% (*w*/*v*) deionized water and then passed consecutively through 0.45 and 0.22 *μ* m syringe filters. To isolate polysaccharides with a molecular weight exceeding 10 kDa, a 10 kDa cassette filter was employed. The polysaccharides were lyophilized for further use.

For nanoparticle preparation, 100 mg of algal polysaccharides was heated in a glass vial for 2 h at temperatures ranging from 120 to 270°C using a muffle furnace in an open-air environment without an inert atmosphere. After cooling to room temperature, the samples were resuspended in deionized water and sonicated for 2 h. They were then centrifuged at 500 × *g* for 30 min to remove aggregates. The supernatant, containing the nanoparticles, was collected for further analysis. Nanoparticles made from SP polysaccharides were labeled S120, S150, S180, S210, S240, and S270, corresponding to their preparation temperatures. UP-based and PP-based nanoparticles were named similarly, following the same temperature-based convention. The prepared nanoparticles were stored in airtight containers at 25°C and 30% relative humidity in a desiccator before use. The molecular weight and monosaccharide composition of algal polysaccharides, and the yield, morphology, particle size, sulfate content, and functional groups of the nanoparticles, were characterized using established methods [18, 24–27].

### 2.3. Evaluation of Antioxidant and Anti-Inflammatory Properties of Algal Polysaccharides-Based Nanoparticles

The antioxidant capacity of the algal polysaccharides and their nanoparticles was measured using the DPPH free radical scavenging and Fe^2+^ chelating assays, as described previously [28, 29]. For the anti-inflammatory test, RAW 264.7 cells (1 × 10^5^ cells/mL) were treated with different concentrations (0–1000 *μ*g/mL) of either algal polysaccharides or their nanoparticles, together with LPS (100 ng/mL), for 24 h. The culture supernatants were collected to measure cytokine and nitric oxide levels using ELISA and Griess reagent kits. Cell viability was also assessed in parallel using the MTT assay [16].

### 2.4. Statistical Analysis

One-way ANOVA was employed to perform the statistical analysis. Post hoc analysis using the least significant difference test was conducted once significant differences were observed. Significance was assigned to results with *p*-values under 0.05.

## 3. Results

### 3.1. Characterization of Algal Polysaccharides and Algal Polysaccharides-Based Nanoparticles

**Figure 1:**
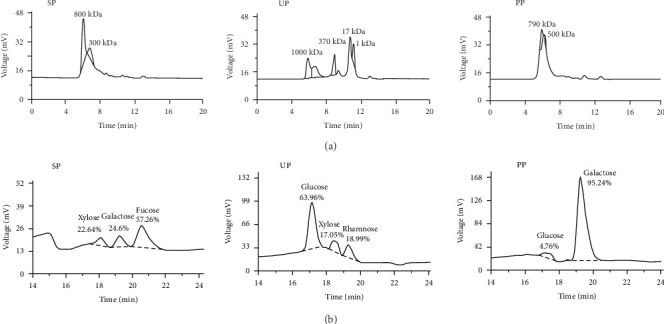
Analysis of molecular weight profiles and monosaccharide constituents of algal polysaccharides. HPLC chromatograms illustrating (A) the molecular weight distribution and (B) the monosaccharide composition of polysaccharide extracts obtained from *Sargassum* (SP), *Ulva* (UP), and *Porphyra* (PP).

The molecular weight distributions of the algal polysaccharides varied among the species. SP exhibited a molecular weight range from 300 to 800 kDa, while UP displayed molecular weight peaks at 1, 17, 370, and 1000 kDa. PP had a molecular weight range from 500 to 790 kDa (Figure 1A). High-performance liquid chromatography (HPLC) for monosaccharide composition analysis showed unique profiles for each polysaccharides. SP contained fucose (57.26%), galactose (24.6%), and xylose (22.64%), UP contained rhamnose (18.99%), xylose (17.05%), and glucose (63.96%), and PP contained galactose (95.24%) and glucose (4.7%; Figure 1B).

**Figure 2:**
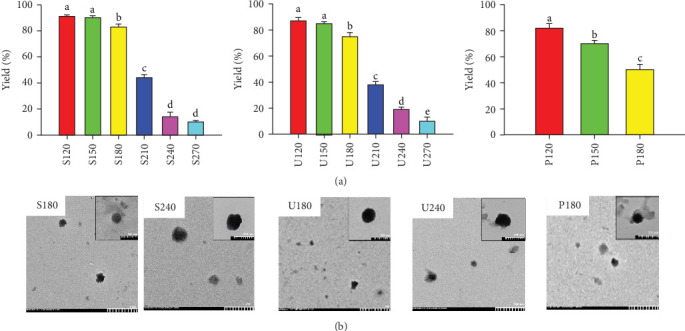
The yield and morphology of algal polysaccharides-based nanoparticles. (A) The yield of nanoparticles is presented as percentages relative to the initial polysaccharides mass. Each value is presented as the average ± standard error of the mean (SEM), derived from three distinct experimental replicates. Statistically significant differences in nanoparticle yield at different preparation temperatures are indicated by different letters (a–e). (B) Representative TEM images of algal polysaccharides-based nanoparticles.

Nanoparticles were prepared from SP, UP, and PP using a dry-heating method. The yield of nanoparticles was found to decrease with increasing temperature (Figure 2A). Notably, PP-based nanoparticles could not be dissolved in water when heated above 180°C due to excessive carbonization, so PP-based nanoparticle preparations were limited to 120, 150, and 180°C. Transmission electron microscopy (TEM) indicated that the nanoparticles possessed a spherical structure and measured under 500 nm in diameter (Figure 2B).

**Figure 3:**
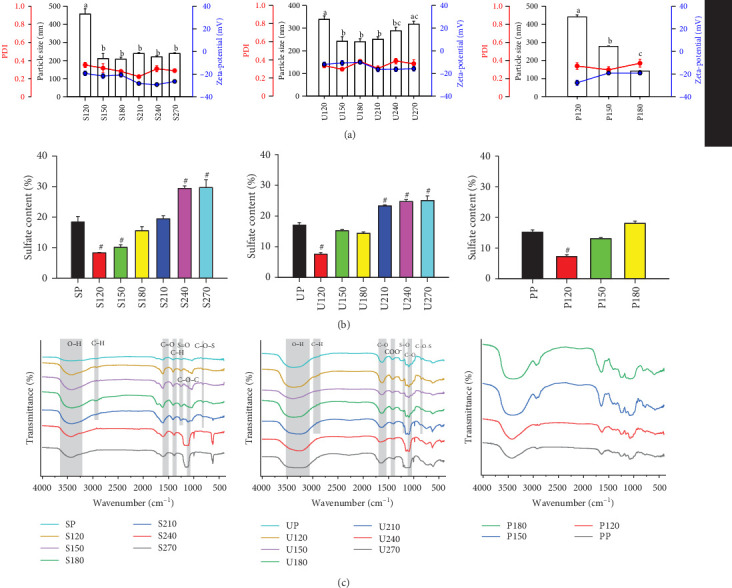
Physical properties, sulfate contents, and functional groups of algal polysaccharides-based nanoparticles. (A) Particle size, PDI, and zeta-potential of algal polysaccharides-based nanoparticles. (B) Sulfate contents. (C) Representative FTIR chromatograms of SP, UP, PP, and their derived nanoparticles. Each value is presented as the average ± standard error of the mean (SEM), derived from three distinct experimental replicates. Statistically significant differences in particle size at different preparation temperatures are indicated by different letters (a–c). A hash symbol (^#^) denotes a statistically significant difference in sulfate content compared to algal polysaccharides.

Dynamic light scattering analysis further confirmed the particle size to be below 500 nm, with a polydispersity index (PDI) ranging from 0.2 to 0.4 and zeta potential values between −5 and −30 mV. Additionally, a noticeable reduction in particle size was observed as the temperature increased up to 150°C, but for UP-based nanoparticles, the size increased above 240°C (Figure 3A). The sulfate content of the nanoparticles varied with the preparation temperature. At 120°C, the sulfate content in the nanoparticles was significantly lower than that of the crude polysaccharides, but it increased at higher temperatures (Figure 3B). Alterations in the functional groups of the nanoparticles were detected through Fourier-transform infrared (FTIR) spectroscopy analysis compared to the original polysaccharides. For SP, the O─H functional group peak at 3419 cm^−1^, C−H peak at 2939 cm^−1^, and sulfate S═O peak at 1258 cm^−1^ were observed, with some signals disappearing or shifting at higher temperatures (Figure 3C). Similar trends were observed for UP and PP-based nanoparticles, where functional group changes occurred, particularly at higher preparation temperatures (Figure 3C).

### 3.2. Antioxidant Activities of Algal Polysaccharides and Algal Polysaccharides-Based Nanoparticles

**Figure 4:**
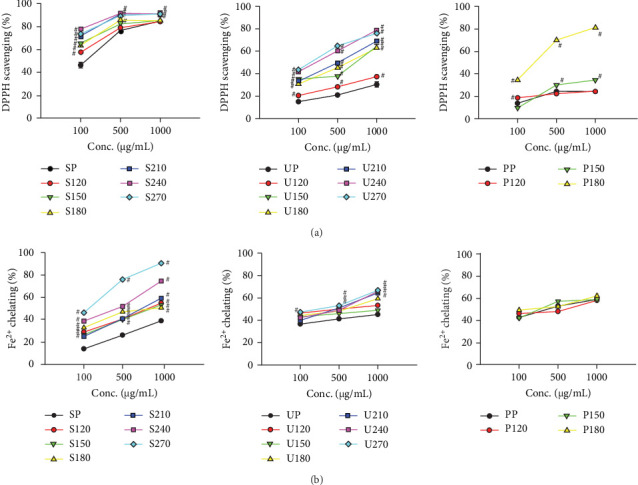
Antioxidant potentials of algal polysaccharides-based nanoparticles. (A) DPPH scavenging and (B) Fe^2+^ chelating activities of SP, UP, PP and their derived nanoparticles at various concentrations. Each value is presented as the average ± SEM, derived from three distinct experimental replicates. A hash mark (^#^) signifies a statistically significant difference when compared to the equivalent concentrations of the polysaccharide samples.

The antioxidant activities of the crude polysaccharides and their corresponding nanoparticles were evaluated using DPPH radical scavenging and Fe^2+^ chelation assays. In the DPPH assay, the radical scavenging activity of crude SP increased with concentration, reaching 45% at 100 *μ*g/mL and 80% at 1000 *μ*g/mL. SP-based nanoparticles exhibited significantly higher scavenging activity compared to crude SP at the same concentrations, with S240 showing the highest activity of approximately 91% at 1000 *μ*g/mL (Figure 4A). These results suggest that nanoparticle formation substantially enhanced the radical scavenging capacity of SP. UP showed weaker DPPH scavenging, with only 30% activity at 1000 *μ*g/mL, yet UP-based nanoparticles demonstrated notable improvement, particularly at higher processing temperatures. U240 and U270 exhibited significantly enhanced activity, with scavenging rates of 78% and 76%, respectively, at 1000 *μ*g/mL, indicating that thermal processing may have contributed to increased antioxidant function. For PP, the crude polysaccharide displayed approximately 24% at 1000 *μ*g/mL. However, P180 nanoparticles reached approximately 80% scavenging at 1000 *μ*g/mL, a statistically significant improvement over the crude PP. These results indicate that nanoparticle formulation, particularly at optimized temperatures, can greatly enhance DPPH scavenging performance.

In the Fe^2+^ chelation assay, crude SP exhibited 38% chelation at 1000 *μ*g/mL, while SP-based nanoparticles demonstrated a clear enhancement in activity. S270, in particular, reached the highest chelation efficiency among SP-derived samples (90% at 1000 *μ*g/mL; Figure 4B). UP in its crude form exhibited 40% chelation, while UP-based nanoparticles demonstrated a modest yet statistically significant increase in chelation capacity, reaching up to 66% at elevated temperatures (U240 and U270). However, this enhancement was less pronounced compared to that observed in SP-derived samples. Interestingly, PP exhibited the highest chelation ability among the crude samples (58% at 1000 *μ*g/mL). However, no significant enhancement was observed upon nanoparticles formation. This may suggest that the Fe^2+^ chelating functional groups in PP were already maximally active in their crude form or that thermal processing did not further enhance (and may have even altered) these groups differently compared to those involved in radical scavenging. These findings indicate that the antioxidant effectiveness of nanoparticles varies by species and processing conditions, with SP-derived nanoparticles consistently showing the greatest enhancement in both radical scavenging and metal chelation activity.

### 3.3. Anti-Inflammatory Potential of Algal Polysaccharides and Algal Polysaccharides-Based Nanoparticles

**Figure 5:**
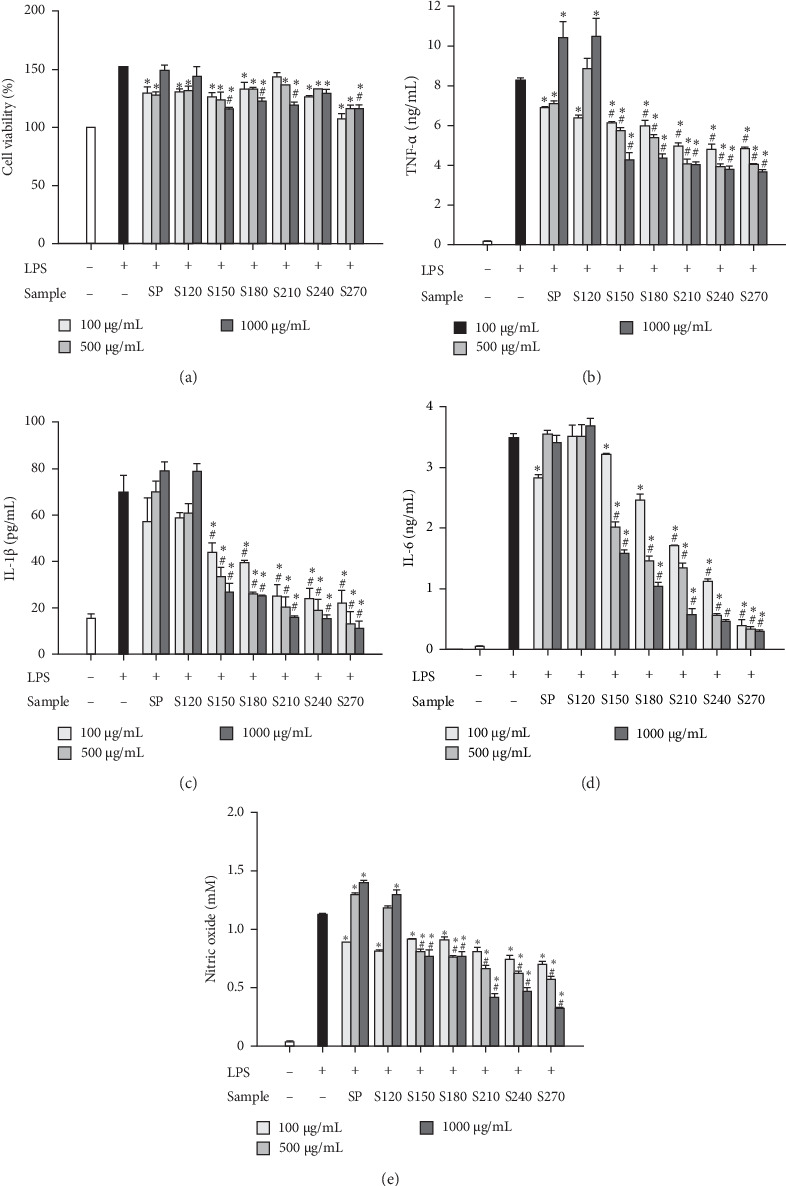
Anti-inflammatory effects of SP and SP-based nanoparticles. RAW 264.7 cells were subjected to treatment with LPS in conjunction with either SP or its nanoparticles, and subsequently (A) cellular viability and the concentrations of (B) TNF-*α*, (C) IL-1*β*, (D) IL-6, and (E) nitric oxide were quantified as detailed in Section 2. Each value is presented as the average ± SEM, derived from three distinct experimental replicates. A hash symbol (^#^) signifies a statistically significant difference when compared to the corresponding concentration of polysaccharides, while an asterisk (*⁣*^*∗*^) signifies a statistically significant difference when compared to LPS alone.

Exposure of RAW 264.7 cells to LPS resulted in notable enhancements in cell viability, alongside an increment of TNF-*α*, IL-1*β*, IL-6 and nitric oxide generation (Figure 5A–E). Treatment with SP or S120 at 100 *μ*g/mL reduced LPS-induced cytokine and nitric oxide production. However, at 1000 *μ*g/mL, the treatment did not reduce, and in some cases, it may have even increased LPS-induced activation and cytokine levels (Figure 5A–E). Notably, SP-based nanoparticles, particularly those prepared at higher temperatures (S150, S180, S210, S240, and S270), significantly alleviated LPS-induced cytokine and nitric oxide production in a manner correlated with concentration (Figure 5B–E). Higher preparation temperatures correlated with enhanced anti-inflammatory potency of the nanoparticles.

**Figure 6:**
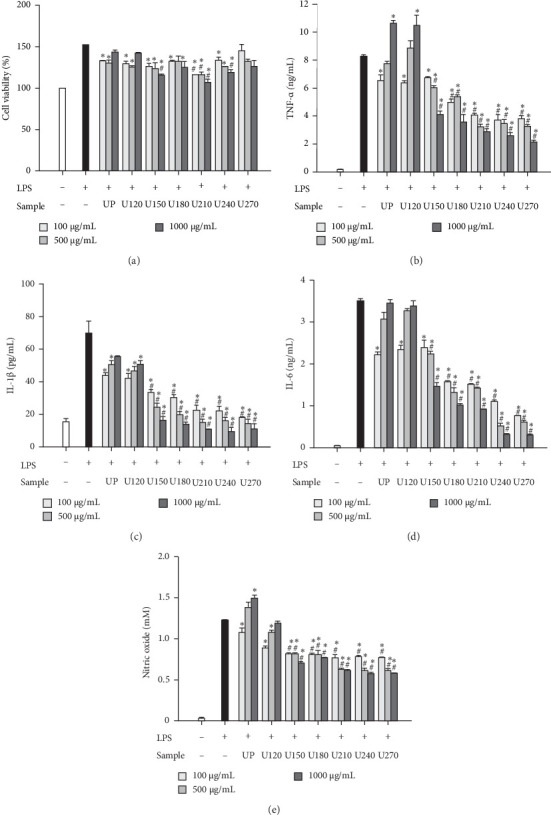
Anti-inflammatory effects of UP and UP-based nanoparticles. RAW 264.7 cells were subjected to treatment with LPS in conjunction with either UP or its nanoparticles, and subsequently (A) cellular viability and the concentrations of (B) TNF-*α*, (C) IL-1*β*, (D) IL-6, and (E) nitric oxide were quantified as detailed in Section 2. Each value is presented as the average ± SEM, derived from three distinct experimental replicates. A hash symbol (^#^) signifies a statistically significant difference when compared to the corresponding concentration of polysaccharides, while an asterisk (*⁣*^*∗*^) signifies a statistically significant difference when compared to LPS alone.

**Figure 7:**
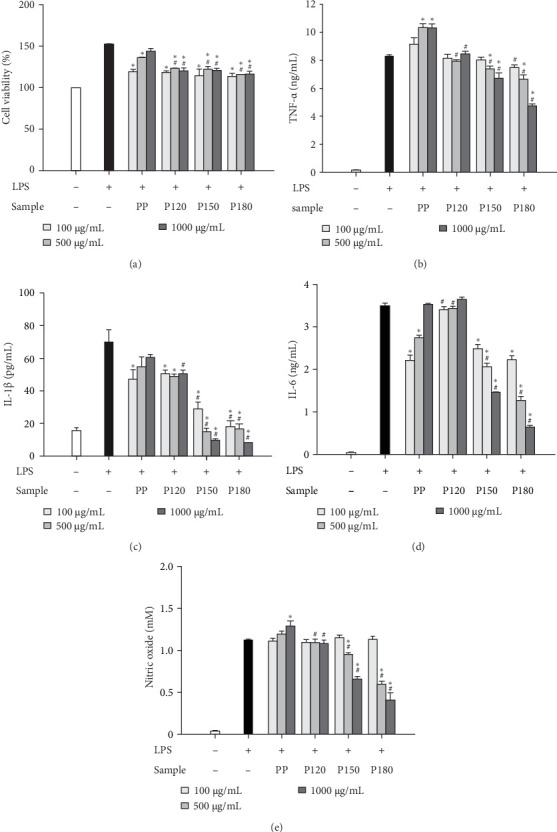
Anti-inflammatory effects of PP and PP-based nanoparticles. RAW 264.7 cells were subjected to treatment with LPS in conjunction with either PP or its nanoparticles, and subsequently (A) cellular viability and the concentrations of (B) TNF-*α*, (C) IL-1*β*, (D) IL-6, and (E) nitric oxide were quantified as detailed in Section 2. Each value is presented as the average ± SEM, derived from three distinct experimental replicates. A hash symbol (^#^) signifies a statistically significant difference when compared to the corresponding concentration of polysaccharides, while an asterisk (*⁣*^*∗*^) signifies a statistically significant difference when compared to LPS alone.

Comparable outcomes were noted in cells following treatment with UP, PP, and their respective nanoparticles. The nanoparticles prepared at temperatures exceeding 150°C demonstrated enhanced inhibition of LPS-induced inflammation, with the anti-inflammatory effects becoming more pronounced as the temperature of nanoparticle preparation increased (Figures 6A–E and 7A–E). These results suggest that algal polysaccharides-based nanoparticles, particularly those prepared at higher temperatures, possess significant anti-inflammatory potential, offering a promising approach to modulating macrophage-driven inflammation.

## 4. Discussion

This research highlights the capability of algal polysaccharides-based nanoparticles to improve antioxidant and anti-inflammatory effects, which are promising for therapeutic applications in nanomedicine. The molecular weight and monosaccharide composition of polysaccharides varied significantly across species, impacting their biological activities The variations at the molecular level were also instrumental in influencing the capacity of these polysaccharides to generate nanoparticles and determine their corresponding bioactivity.

The dry-heating method used to prepare nanoparticles produced spherical particles with sizes ranging from 100–500 nm, which are known optimal for cellular uptake and efficient delivery [30]. Notably, the preparation temperature had a significant effect on physicochemical properties, and all of which are crucial for their biological activities. For example, preparation of carbonized polymer nanosheets at 180°C leads to the formation of well-structured 2D nanosheets with enhanced *π* →*π* *⁣*^*∗*^ and n→*π* *⁣*^*∗*^ transitions. The D and G bands in Raman spectra become sharper, indicating improved graphitization, while the zeta potential decreases from −46 mV at 180°C to −11 mV at 240°C, reflecting the loss of functional groups. At a temperature of 180°C, carbonized polymer nanosheets demonstrated exceptional bacterial adsorption capabilities, effectively eliminating over 98% of *Vibrio parahaemolyticus* within a 2-h period, with their efficacy sustained for up to 24 h. However, further increasing the temperature to 210–240°C results in excessive carbonization and aggregation, reducing antimicrobial efficiency [31]. In the case of polyphenolic carbonized nanogels, synthesis at 150–180°C produces gel-like crosslinking polymers, while higher temperatures between 210 and 300°C induce the formation of graphene-like structures. The particle size decreases with increasing temperature, reaching 52.0 nm at 270°C, which corresponds to the highest anticoagulation activity (526-fold higher than untreated alginate) due to the formation of polyphenolic structures that strongly bind to thrombin. However, at 300°C, over-crosslinking and aggregation occur, leading to an increase in particle size (198.2 nm) and a reduction in bioactivity [32]. Accordingly, 180°C is optimal for carbonized polymer nanosheets to achieve enhanced antimicrobial performance, while 270°C is ideal for polyphenolic carbonized nanogels to maximize anticoagulation activity. Excessive temperatures in both cases lead to structural aggregation and diminished bioactivities [31, 32]. In the current study, PP-based nanoparticles showed poor solubility when heated above 180°C due to excessive carbonization, emphasizing the need to control temperature during nanoparticle synthesis. Additionally, the sulfate content varied with temperature, indicating that temperature control is critical for optimizing the properties of nanoparticles for specific therapeutic applications.

Despite prior research highlighting the anti-inflammatory attributes of nanomaterials derived from algal polysaccharides, these formulations have typically involved the incorporation of additional synthetic or natural polymers rather than being composed exclusively of algal polysaccharides. For example, ulvan-based nonwoven nanofibrous patches blended with polyethylene oxide have shown significant efficacy in promoting wound healing and reducing skin inflammation over a 21-day period in patients undergoing cryosurgery [33]. Ulvan-coated selenium nanoparticles can alleviate acute colitis in mice. This attenuation is achieved by reducing body weight loss, lessening damage to colonic tissue, and diminishing macrophage infiltration. These beneficial therapeutic outcomes were linked to their capacity to modulate crucial pro-inflammatory cytokines [34]. Similarly, orally administered PLGA nanoparticles encapsulating resveratrol, chitosan and alginate effectively acted on inflamed colonic tissues and improved inflammatory markers in mice with colitis [35]. In vitro studies further support the anti-inflammatory potential of algal polysaccharide-based systems. Chitosan–alginate nanoparticles significantly reduced *Propionibacterium acnes*-induced IL-12p40 and IL-6 generation in monocytes and HaCaT keratinocytes, respectively [36]. In addition, fucoidan/chitosan nanoparticles loaded with methotrexate were found to suppress pro-inflammatory cytokine expression in activated human monocytes [37]. Despite these advances, current research has largely focused on nanomaterials composed of mixed biopolymers. To date, there have been no reports specifically investigating the bioactivity of nanomaterials derived solely from red algal polysaccharides. This study demonstrates that the algal polysaccharides alone can form stable nanoparticles without the need for carrier polymers, underscoring the simplicity and novelty of the employed system.

In this study, antioxidant properties of the nanoparticles were assessed employing both the DPPH free radical scavenging assay and the Fe^2+^ chelation assay. SP-based nanoparticles, particularly those heated at higher temperatures (S240 and S270), showed the most significant improvement in antioxidant activity. This enhancement likely resulted from structural modifications during the dry-heating process, which may increase the availability of functional groups involved in antioxidant action [38]. In contrast, UP-based nanoparticles exhibited a more moderate increase in antioxidant activity, and PP-based nanoparticles showed the weakest effects. These results suggest that antioxidant potential is both species-specific and dependent on preparation temperature. In the Fe^2+^ chelation assay, SP-based nanoparticles, particularly those heated at 270°C, demonstrated significantly improved metal ion chelation, a mechanism essential for preventing oxidative stress-related diseases [39]. The weaker enhancement observed for UP- and PP-based nanoparticles further supports the notion that the antioxidant potential of algal polysaccharide-based nanoparticles is influenced by their chemical characteristics [40]. The anti-inflammatory efficacy was evaluated using LPS-treated RAW 264.7 cells, a commonly used model for macrophage-mediated inflammation. SP-based nanoparticles, especially those prepared at higher temperatures, effectively diminished the generation of pro-inflammatory cytokines and nitric oxide induced by LPS, with the extent of inhibition depending on the concentration. These findings align with previous studies suggesting that polysaccharides from marine algae possess immunomodulatory properties that can suppress inflammation by inhibiting macrophage activation [14]. The enhanced anti-inflammatory effects of nanoparticles prepared at higher temperatures suggest that heat treatment induces structural changes, such as modifications in surface charge, size, and bioactivity, that contribute to improved anti-inflammatory properties. Similar effects were observed for UP- and PP-based nanoparticles, especially those prepared at temperatures above 150°C. These findings further confirm that algal polysaccharide-based nanoparticles, particularly those synthesized at higher temperatures, show potential as effective anti-inflammatory agents for therapeutic development.

The enhanced bioactivities of algal polysaccharide-based nanoparticles are closely linked to structural modifications induced by thermal processing. FTIR analysis showed that increasing preparation temperatures led to reduced or shifted O─H, C─H, and S═O peaks—indicative of dehydration, bond cleavage, and possible formation of conjugated or aromatic structures. Such transformations likely enhance electron-donating ability, explaining the improved DPPH radical scavenging [41]. Additionally, higher sulfate content in SP and UP nanoparticles processed at elevated temperatures may result from selective degradation or exposure of sulfate-enriched regions. These sulfate groups are well-known to enhance antioxidant and anti-inflammatory functions through interaction with radicals, metal ions, and immune mediators [42]. The nanoscale size of the particles (<500 nm) further increases surface area and bioavailability, consistent with studies showing enhanced functional activity of polysaccharide nanoparticles [43]. However, excessive heating, as seen in PP nanoparticles above 180°C, led to carbonization and loss of solubility, underscoring the need for an optimized thermal window to balance structural enhancement with material integrity.

Notably, nanoparticles prepared at lower temperatures (e.g., 120°C) exhibited diminished or even reversed anti-inflammatory effects at higher concentrations. This may be attributed to their structural features remaining more similar to those of the native polysaccharides, potentially limiting their bioactivity. In contrast, nanoparticles generated at higher temperatures (≥150°C) demonstrated a more consistent and pronounced concentration-dependent anti-inflammatory response. This suggests that structural modifications induced by thermal treatment enhance biological interactions. However, at high concentrations, a plateau or reduction in efficacy may occur due to saturation of cellular uptake mechanisms [44]. Furthermore, nanoparticle aggregation at elevated doses could reduce bioavailability or interfere with receptor binding, thereby attenuating anti-inflammatory effects [45]. Although cytotoxicity assays confirmed cell viability up to 1000 *μ*g/mL, subtle stress responses or unintended immune activation at higher concentrations cannot be entirely excluded [46]. While this study demonstrates enhanced antioxidant and anti-inflammatory properties of algal polysaccharides-based nanoparticles in vitro, several important limitations must be acknowledged. The primary limitation of this study is its reliance on cell-based assays, with in vivo efficacy, pharmacokinetics, biodistribution, and long-term safety yet to be investigated. These parameters are essential for fully assessing the therapeutic potential and systemic compatibility of the nanoparticles [47]. To address these gaps, future studies will incorporate animal models to evaluate the translational relevance of our findings under physiologically realistic conditions. In addition, although clear correlations were observed between processing temperature, nanoparticle structure, and biological activity, the precise mechanisms of action remain to be elucidated. Specifically, how these nanoparticles interact with macrophages or neutralize reactive oxygen species is not yet fully understood. Future investigations will focus on elucidating these mechanisms through molecular pathway analysis, receptor-binding studies, and cellular uptake assays. Of particular interest is determining whether the anti-inflammatory effects are mediated through modulation of key signaling pathways such as NF-*κ*B or MAPK [48]. Furthermore, species-specific differences observed among SP-, UP-, and PP-based nanoparticles suggest that algal source composition significantly influences nanoparticle structure and function. This warrants further exploration to optimize each formulation based on its intrinsic polysaccharides characteristics. Finally, while this study employed multiple techniques for nanoparticle characterization, including molecular weight distribution, functional group analysis, and surface morphology, additional structural analyses, such as Raman spectroscopy, X-ray photoelectron spectroscopy, or elemental composition analysis, could provide deeper insights into thermal degradation, graphitization, or loss of functional groups at elevated temperatures. These will be incorporated in future studies to strengthen understanding of nanoparticle structure–activity relationships. Together, these efforts will be critical to fully unlocking the biomedical potential of algal polysaccharides-based nanoparticles as safe, natural, and biocompatible agents for managing oxidative stress and inflammation-related conditions.

## 5. Conclusion

These results underscore the importance of the algal polysaccharides source and the temperature used during preparation in determining the characteristics and biological activities of the nanoparticles. Notably, nanoparticles prepared at higher temperatures exhibited enhanced antioxidant and anti-inflammatory activities, making them promising for treating oxidative stress and inflammation-related diseases. These findings align with prior investigations concerning polysaccharide-based and carbon nanomaterials, underscoring the importance of temperature control. Future research should explore the mechanisms behind these bioactivities and assess the in vivo safety and efficacy of these nanoparticles.

## Data Availability

The data that support the findings of this study are available from the corresponding author upon reasonable request.
